# Physical Realization of von Neumann Lattices in Rotating Bose Gases with Dipole Interatomic Interactions

**DOI:** 10.1038/srep31801

**Published:** 2016-08-22

**Authors:** Szu-Cheng Cheng, Shih-Da Jheng

**Affiliations:** 1 Dept. of Optoelectric Physics, Chinese Culture University, Taipei 11114, Taiwan, ROC; 2 Institute of Physics, National Chiao Tung University, Hsinchu 30010, Taiwan, ROC

## Abstract

This paper reports a novel type of vortex lattice, referred to as a bubble crystal, which was discovered in rapidly rotating Bose gases with long-range interactions. Bubble crystals differ from vortex lattices which possess a single quantum flux per unit cell, while atoms in bubble crystals are clustered periodically and surrounded by vortices. No existing model is able to describe the vortex structure of bubble crystals; however, we identified a mathematical lattice, which is a subset of coherent states and exists periodically in the physical space. This lattice is called a von Neumann lattice, and when it possesses a single vortex per unit cell, it presents the same geometrical structure as an Abrikosov lattice. In this report, we extend the von Neumann lattice to one with an integral number of flux quanta per unit cell and demonstrate that von Neumann lattices well reproduce the translational properties of bubble crystals. Numerical simulations confirm that, as a generalized vortex, a von Neumann lattice can be physically realized using vortex lattices in rapidly rotating Bose gases with dipole interatomic interactions.

Rotating gases experience a centrifugal force, referred to as the Coriolis force, in the rotating frame. The force acting on two-dimensional (2D) rotating gases with a specific rotational frequency is similar to the Lorentz force experienced by charged particles in a uniform magnetic field. The wave functions and energy levels of a particle can be solved by selecting a particular gauge for the magnetic field. Abrikosov^1^ constructed a macroscopic wave function to predict the existence of a vortex lattice that contains a single flux quantum per unit cell. The wave function is expanded over periodic Landau wave functions in the lowest Landau level (LLL)^2^. This lattice is referred to as the Abrikosov lattice and can be found in type-II superconductors^3^,^4^, superfluid helium^5^,^6^, Bose-Einstein condensates^7^,^8^, ultracold fermion superfluids^9^, and dark matter condensates^10^. Generally, Abrikosov lattices are energetically stable in physical systems with contact interactions^11^. Bose-Einstein condensates with long-range interactions, such as dipolar interactions^12^,^13^, dipole-blockaded interactions^14^,^15^,^16^ or Rydberg-dressed interactions^1^,^17^,^18^ have recently been studied and supersolid droplet crystals have been discovered in a dipole-blockaded gas^14^,^15^,^16^. The dipole blockade is used to control the generation of collective excitations and manipulate the entanglement of these excitations for the processing of quantum information^19^,^20^.

Rotating dipolar quantum gases have been shown to contain a number of novel crystal phases^13^. Wigner crystals and bubble crystals occur in rapidly rotating dipolar Fermi and Bose gases, respectively. Zhang and Zhai used Abrikosov wave functions to compare the ground-state energies of vortex lattices with different symmetries in a fast rotating dipolar condensate^21^. They concluded that the energetically stable phases of vortex lattices in a fast rotating dipolar condensate are triangular, square, or strip-shaped. The results found by Zhang and Zhai^21^ were consistent with the calculations done by Cooper *et al*. when the dipole-dipole interaction was weak^22^. However, in the case of strong dipolar interactions, particles were shown to cluster into bubble states^22^, which are labeled according to the number of vortices *q* associated with each bubble within a triangular lattice, where *q* is an integral number. This bubble phase was not observed in the fast rotating dipolar condensate in the study by Zhang and Zhai^21^. Nonetheless, its existence was later confirmed in the simulation of rotating Rydberg-dressed Bose gases^17^. This apparent contradiction indicates that the model of vortex lattices is incomplete with regard to vortex lattices in Bose gases with long-range interactions. It is therefore important to develop a model of vortex lattices with the same lattice structure as the Abrikosov lattice in cases where the lattice contains a single quantum flux per unit cell. This model would also have to explain the clustering of particles in the form of bubble crystals in which the number *q* of flux quanta per unit cell exceeds 1 (*q* > 1).

In this paper, we first demonstrate that bubble crystals are the stable states of rapidly rotating Bose gases with dipole interatomic interactions (RRDGs) by solving the corresponding Gross-Pitaeviskii equation numerically. Then, we develop a model of vortex lattices, referred to as a von Neumann lattice^23^. The von Neumann lattice is a lattice in a gauge field, abbreviated as vN_*q*_, with *q* flux quanta per unit cell. A vN_1_ lattice possesses some common physical properties as an Abrikosov lattice. The bubble crystals, discovered in rapidly rotating Bose gases with long-range interactions, are compared with vN_*q*_ lattices with *q* > 1. We show that bubble crystals can be approximately represented by vN_*q*_ lattices with *q* > 1. Using a vN_*q*_ lattice as the ansatz of the ground state of RRDGs, we can calculate the ground-state energies of RRDGs and devise a phase diagram of bubble crystals in RRDGs. The validity of the phase diagram was also confirmed using numerical simulations.

## Numerical Simulations and Results of Bubble Crystals

Rotating 2D quantum gases with rotational frequency Ω are affected by gauge field **A**, 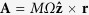 for a symmetric gauge. Here, *M* refers to the mass of an atom, ***r*** = (*x, y*) is a 2D coordinate and 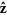 is the direction perpendicular to the 2D plane. The kinetic Hamiltonian *H*_0_ of RRDGs is given by *H*_0_ = (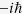
∇−***A***)^2^/2*M*. We assume that the state of RRDGs is a condensate which is governed by the Gross-Pitaeviskii equation of RRDGs. The Gross-Pitaeviskii equation of RRDGs is written as follows:


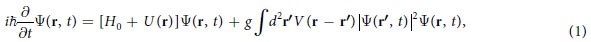
where *H*_0_ is the kinetic Hamiltonian and *U*(***r***) = *M*(*ω*^2^−Ω^2^)*r*^2^/2 is the trapping potential with ω as the radial trapping frequency. When the macroscopic wave function Ψ(***r**, t*) is normalized by ∫|Ψ(***r**, t*)|^2^*d*^2^***r*** = 1, the coupling constant *g* = *ND*, where *N* and *D* are the number of particles and the dipole interaction strength, respectively. *V*(**r**) in Eq. (1) is the dipole-interatomic potential given by^14^


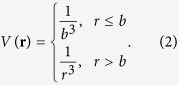
where *b* is the blockade radius.

**Figure 1:**
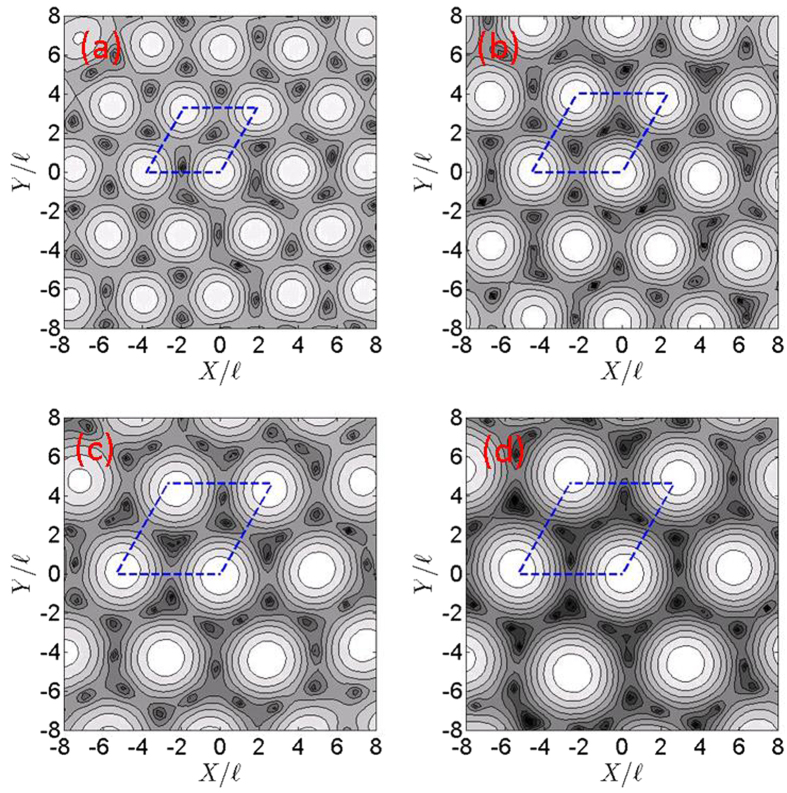
Contour plots of log-scaled particle density distributions of bubble crystals. The low-density regime marked as a black area indicates the location of a vortex and the bright area is the high-density regime. The blue dashed lines outline the area of a unit cell. The density distributions of bubble crystals for blockade radius 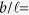
1.88, 2.28, 2.68 and 3.02 are presented in diagrams (**a–d**), respectively. The average lattice constants for diagrams (**a–d**) are 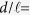
3.83, 4.48, 5.18 and 5.80, respectively.

With a fixed value for *b* and Ω/*ω* = 0.9, the structure of vortex lattices of RRDGs was numerically obtained by solving Eq. (1) (see Supplementary for the simulation method). We adopted an interaction strength of *g* = 1500. Figure 1 presents the density distributions of vortex lattices obtained from numerical simulations. We clarified the vortex distribution by drawing the log-density distributions of bubble crystals in Fig. 1. The blue dashed lines outline the area of a unit cell. These distributions for blockade radius 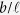 = 1.88, 2.28, 2.68 and 3.02 are presented in diagrams 1(a), 1(b), 1(c) and 1(d), respectively. Here 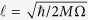 is the magnetic length. We find that atoms in a vortex lattice are clustered into a triangular bubble crystal and surrounded by vortices. The average lattice constants for diagrams 1(a), 1(b), 1(c) and 1(d) are 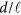 = 3.83, 4.48, 5.18 and 5.80, respectively. There is a trend that the lattice constant of a bubble crystal is increasing with blockade radius *b*. Based on the vortex distribution in the unit cells outlined by blue dashed lines in Fig. 1, we can count the number *q* of flux quanta per unit cell. Diagrams 1(a), 1(b), 1(c) and 1(d) have *q* = 2, 3, 4, and 5 per unit cell, respectively. The number of flux quanta per unit cell is increased as the blockade radius *b* becomes larger. Triangular bubble crystals with an integral number of flux quanta per unit cell are energetically favorable in the regime of a larger blockade radius of RRDGs.

We discussed the physical properties of bubble crystals obtained by using the imaginary-time-propagation method to the Gross-Pitaeviskii equation of RRDGs. The case of a vortex lattice with *q* = 1 corresponds to the Abrikosov lattice, while cases with *q* > 1 go beyond the Abrikosov model. In this report, we will show that a von Neumann lattice, which is a subset of coherent states and exists periodically in the physical space, owns the same physical properties as bubble crystals. We will also compare the structure of vN_*q*_ lattices with bubble crystals to see whether bubble crystals can be described by vN_*q*_ lattices.

### Model of von Neumann Lattices

Let us introduce the complex number 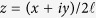 and its complex conjugate 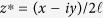, where 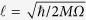 is the magnetic length. The corresponding derivatives of *z* and z^*^ are 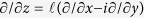 and 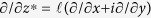. We then represent two pairs of harmonic oscillator operators as^2^: *a* = −*i*(*z* + ∂/∂z*)/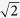, *a*^+^ = *i*(z*−∂/∂*z*)/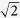, *c* = (z* + ∂/∂*z*)/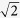 and *c*^+^ = (*z*−∂/∂z*)/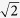. Operators *a* and *c* are inter-Landau-level and intra-Landau-level annihilation operators, respectively. While *a*^+^ and *c*^+^ are creation operators of *a* and *c*. These harmonic oscillator operators satisfy the following commutation relationship: [*a, a*^+^] = [*c, c*^+^] = 1 and [*a, c*] = [*a, c*^+^] = 0. The kinetic Hamiltonian of the condensate can be rewritten as *H*_0_ = 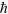
Ω (*a*^+^*a* + *aa*^+^).

Using *a* and *c* operators, we define the vacuum state 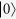 of the system in order to satisfy 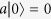, 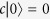 and 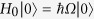. The wave function of the vacuum state^2^, given by 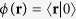, is a Gaussian function of 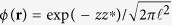. Based on this vacuum state, we then consider the coherent states 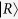 as 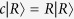 and 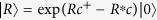, where *R* is a complex number. The set of all coherent states is overcomplete in cases where *R* is a continuous complex number. The overcompleteness means that a complete subset belongs to one of the subsets of the coherent states. Von Neumann^23^ found that a set of periodic coherent states, called the von Neumann lattice, is complete if *R* belongs to a set of lattice points with discrete values *R*_*mn*_, where *m* and *n* are integers. The uncertainty relationship is inherent to the lattice structure. This lattice is a fundamental element in quantum mechanics and has many applications^24^,^25^. The von Neumann procedure makes it possible to construct a complete set of coherent states in the LLL^26^,^27^.

In the following, we consider a von Neumann lattice with primitive vectors ***a***_1_ = *d*(1, 0) and ***a***_2_ = *d*(*u, v*), where *d* is the lattice constant and *u* and *v* are the geometric parameters of a lattice. We take 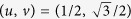 and (0, 1) in the case of triangular and square lattices, respectively. The area of a unit cell in the lattice is *vd*^2^. A von Neumann lattice with a single flux quantum per unit cell revealed a number of applications to deal with problems associated with many electrons^27^. In this study, we extended the von Neumann lattice to one with an integral number of flux quanta per unit cell. Let *ϕ* = *h*/*M* be a flux quantum of circulation. The area of a unit cell is then quantized to *qϕ*/2 Ω, namely, *vd*^2^ = *qϕ*/2 Ω. Lattice points of the von Neumann lattice are given by ***R***_*mn*_ = *m**a***_1_ + *n**a***_2_ or 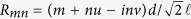. The coherent state at a lattice point *R*_*mn*_ is 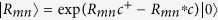. Let 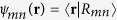 be the wave function of this coherent state in the LLL. Thus, we find that 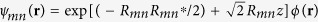, which is the translation of *ϕ*(***r***) from the origin to the lattice site ***R***_*mn*_ and has Gaussian form. We can express *ψ*_*mn*_(***r***) as follows:^2^


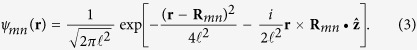
Due to the relationship *vd*^2^ = *qϕ*/2 Ω, the lattice constant increases with an increase in the number of flux quanta per unit cell. The fill factor *f* of the system is given by *f* = *N*_*c*_/*q*, where *N*_*c*_ is the number of particles per unit cell. If *N*_*c*_ < *q*, then the system is in the fractional quantum Hall regime and the quantum-Hall liquid has an energy level below the vortex-lattice state^28^. In this report, we consider the case in which *N*_*c*_ > *q*; i.e., *f* > 1. In the case of *f* > 1, the vortex-lattice state is believed to be energetically stable^8^,^11^.

The coherent state in Eq. (3) represents a cluster of bosonic atoms residing at a lattice site. Let Ψ_*q*_(***r***) be the macroscopic wave function of a vortex lattice with *q* flux quanta per unit cell. Owing to the completeness of the coherent state *ψ*_*mn*_(***r***), we can expand Ψ_*q*_(***r***) in terms of a von Neumann lattice, as follows:


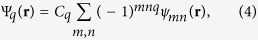
where *C*_*q*_ is a normalized constant determined by the number of particles in the system. Note that 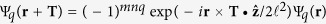, where ***T*** = *m**a***_1_ + *n**a***_2_. Therefore Ψ_*q*_(***r***) is not a periodic function, but rather a quasi-periodic function characterized by the vortex-lattice structure^29^. The density distribution *ρ*_*q*_(***r***), defined by *ρ*_*q*_(***r***) = |Ψ_*q*_(***r***)|^2^, is a periodic function of the lattice; i.e., *ρ*_*q*_(***r*** + ***T***) = *ρ*_*q*_(***r***). Kwasigroch and Cooper used the wave function Ψ_1_(***r***) to study the properties of vortex lattices associated with single vortices in rapidly rotating ultra-cold atomic gasses^25^. We generalized the wave functions of vortex lattices to lattices with *q* flux quanta per unit cell.

The density distributions of vN_1_ lattices show the same geometrical structure as Abrikosov lattices (see Supplementary Fig. S1). Both vN_1_ and Abrikosov lattices possess some common physical properties. To demonstrate these common properties, we considered vN_1_ lattices in rapidly rotating Bose gases with contact interactions. The parameter *β*_*A*_, referred to as the inverse participation ratio, is used to determine the strength of the nonlinear effect and ground-state energy of various vortex structures^1^,^11^. In the case of vN_*q*_ lattices, this inverse participation ratio is denoted as *β*_*q*_, which depends on *qϕ* flux quanta passing through a unit cell and is defined by *β*_*q*_ = 〈|Ψ_*q*_|^4^〉/〈|Ψ_*q*_|^2^〉^2^, where 〈*F*〉 is the average of function *F* in a unit cell. The inverse participation ratio *β*_*q*_ increases as Ψ_*q*_(***r***) becomes increasingly localized and peaked^30^. We then calculated the inverse participation ratio for various vN_*q*_ lattices to enable a comparison with the *β*_*A*_ values of Abrikosov lattices. We obtained 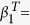
1.1596 and 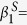
1.1803 for triangular and square vN_1_ lattices, respectively. These values are equivalent to the *β*_*A*_ values found in type-II superconductors^30^ and Bose-Einstein condensates^25^,^31^. In addition, we were able to determine the ground state energies of vN_*q*_ lattices with *q* > 1 (see Supplementary Table 1). Here, we only show 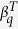 values of these lattices with a triangular structure: 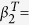
1.3390, 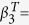
1.6015 and 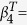
2.0355. The fact that these 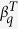 values increase with *q* and exceed the value of 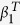 indicates that the Abrikosov lattice is the lowest-energy state in systems with contact interactions^1^. For systems with long-range interactions between particles, it remains an open question as to whether Abrikosov lattices are still energetically favorable^17^,^22^.

### Vortex Lattice Pattern of von Neumann Lattices

**Figure 2:**
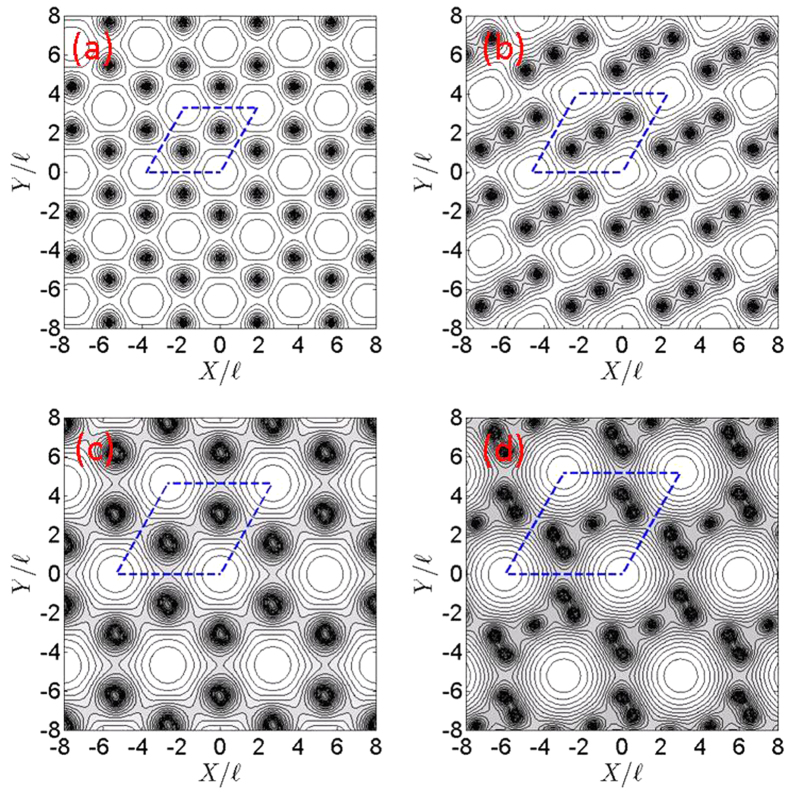
Contour plots of log-scaled particle density distributions of triangular von Neumann lattices. The low-density regime marked as a black area indicates the location of a vortex and the bright area is the high-density regime. The blue dashed lines outline the area of a unit cell. The diagrams in (**a,b**) present the log-density distributions of triangular vN_2_ and vN_3_ lattices, respectively. The diagrams in (**c,d**) present the log-density distributions of triangular vN_4_ and vN_5_ lattices, respectively. The lattice constants for triangular vN_2_, vN_3_, vN_4_ and vN_5_ lattices are 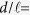
3.81, 4.67, 5.39 and 6.02, respectively.

We clarified the vortex distribution by drawing the log-density distributions, 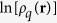, of the triangular vN_*q*_ lattices in Fig. 2. The blue dashed lines also outline the area of a unit cell. Atoms in a vN_*q*_ lattice with a higher number of flux quanta per unit cell are clustered periodically and surrounded by vortices. Clustered atoms form a triangular lattice. vN_*q*_ lattices then appear as bubble crystals^22^ or the supersolids^17^ found in rapidly rotating dipolar and Rydberg-dressed gases. The lattice constants for triangular vN_2_, vN_3_, vN_4_ and vN_5_ lattices are 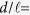
3.81, 4.67, 5.39 and 6.02 in diagrams 2(a), 2(b), 2(c) and 2(d), respectively. Due to the finite-size effect of the trapping potential *U*(**r**) given in Eq. (1), the average lattice constants of the bubble crystals shown in Fig. 1 are slightly smaller than the lattice constants of corresponding infinite vN_*q*_ lattices.

**Figure 3:**
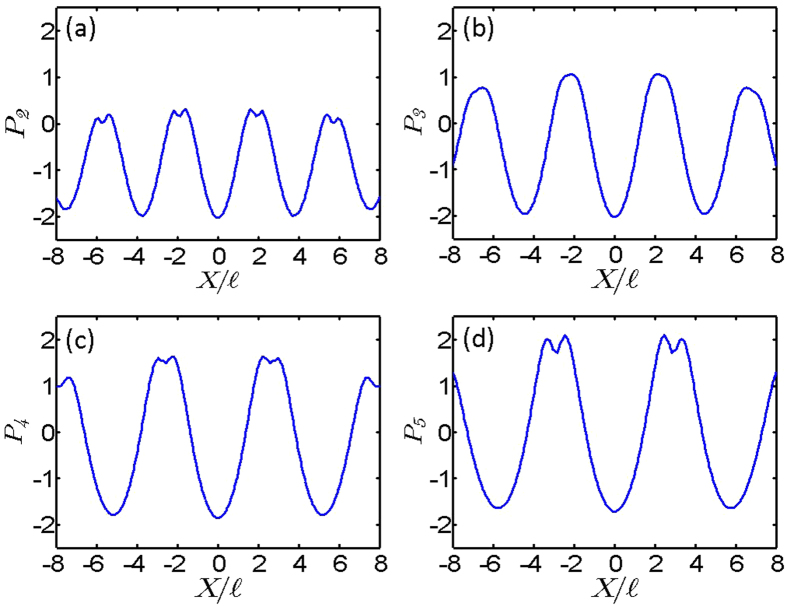
Quantum pressure distributions of bubble crystals. The blue-solid lines represent the quantum pressure distributions along the horizontal axis at 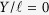 of bubble crystals. The quantum pressure distributions of bubble crystals with blockade radius 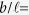
1.88, 2.28, 2.68 and 3.02 are presented in diagrams 3(a), 3(b), 3(c) and 3(d), respectively.

We believe that the quantum pressure *F*_*q*_(***r***) associated with atoms becomes stronger as the number of clustered atoms increases in number according to *b*, where *F*_*q*_(***r***) = *P*_*q*_(***r***)
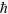
2/2*M* and 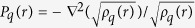 is the dimensionless quantum pressure^32^,^33^. To check the correctness of this belief, we used the existing numerical data to calculate *P*_*q*_(***r***) for various blockade radius. In Fig. 3, we plot the distributions of *P*_*q*_(***r***) along the horizontal axis at 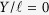, as shown in Fig. 1. We observed that the maximum values of *P*_*q*_(***r***) are 0.31, 1.05, 1.63, and 2.09 in diagrams 3(a), 3(b), 3(c) and 3(d) for bubble crystals with blockade radius 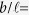
1.88, 2.28, 2.68 and 3.02, respectively. The quantum pressure is negative at a lattice site and becomes positive in between lattice sites. The strength of the quantum pressure associated with atoms is stronger as the blockade radius *b* increases. The number *q* of flux quanta per unit cell depends on the strength of the quantum pressure. An increase in quantum pressure would squeeze a larger number of flux quanta into a unit cell.

Ordinarily, the number of single vortices per unit cell is equal to the number *q* of flux quanta per unit cell. This trend does not apply to the triangular vN_4_ lattice due to its geometrical structure. The square vN_4_ lattice has 4 vortices per unit cell, whereas the triangular vN_4_ lattice has 2 vortices with double flux quanta per unit cell (see Supplementary Fig. S2). These vortex distributions exhibit the same pattern as triangular bubbles, which possess multiple flux quanta associated with each bubble in rapidly rotating dipolar gases^22^.

**Figure 4:**
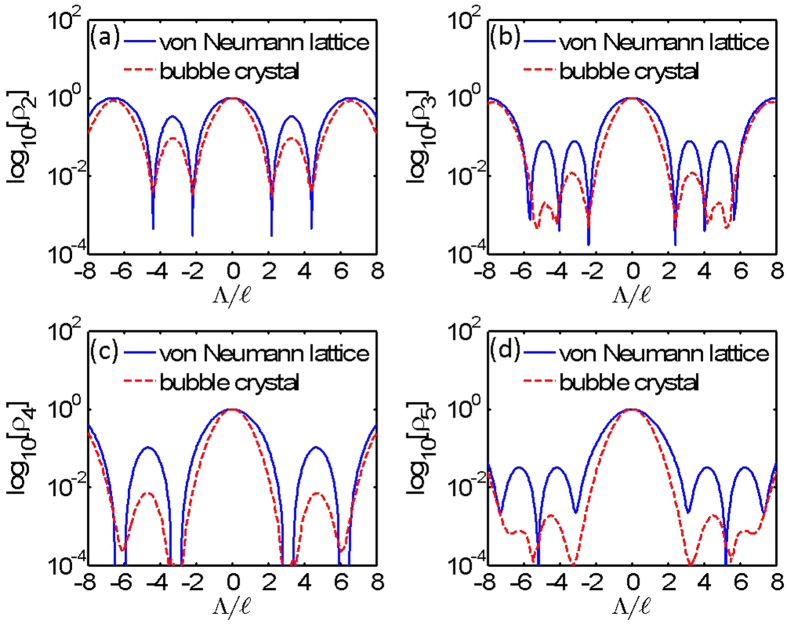
Normalized and log-scaled density distributions of bubble crystals and von Neumann lattices. The red-dashed and blue-solid lines represent the density distributions for bubble crystals and vN_*q*_ lattices, respectively. *Λ* is the coordinate along the diagonal direction of blue-dashed unit cells shown in Figs 1 and 2. The density distributions of lattices with flux quanta *q* = 2, 3, 4 and 5 per unit cell are presented in diagrams 4(a), 4(b), 4(c) and 4(d). 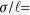
0.82 (1.14), 0.80 (1.12), 0.83 (1.17), and 0.86 (1.17) for bubble crystals (vN_*q*_ lattices) in diagrams (**a–d**), respectively.

To see whether bubble crystals can be represented by vN_*q*_ lattices, we compare the normalized and log-scaled density distributions of bubble crystals and vN_*q*_ lattices along the diagonal direction of unit cells shown by the blue dashed lines in Figs 1 and 2. These distributions are shown in Fig. 4. The density distributions of lattices with flux quanta *q* = 2, 3, 4 and 5 per unit cell are shown in diagrams 4(a), 4(b), 4(c) and 4(d), respectively. The dashed-red and solid-blue lines in Fig. 4 are the density distributions of bubble crystals and vN_*q*_ lattices, respectively. The locations of maxima and minimum densities of bubble crystals are consistent with vN_*q*_ lattices. But, the quantity σ, referred to as a peak width, for the density in a unit cell of a bubble crystal is smaller than the σ of a von Neumann lattice. We find that 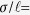
0.82 (1.14), 0.80 (1.12), 0.83 (1.17), and 0.86 (1.17) for bubble crystals (vN_*q*_ lattices) in diagrams 4(a), 4(b), 4(c) and 4(d), respectively. We think that this smaller σ for a bubble crystal is caused by the pressure of the trapping potential. No such pressure is added to vN_*q*_ lattices. Except the peak width being different for bubble crystals and vN_*q*_ lattices, the structure of vN_*q*_ lattices matches that of bubble crystals obtained in numerical simulations. While the von Neumann lattices reproduce well the translational properties of the bubble crystals, there still exists some discrepancy between the wave functions that remains to be clarified.

### Vortex Lattices of Rotating Gases with Long-range Interactions

We know that vN_*q*_ lattices are good representations of bubble crystals occurred in RRDGs. The ground-state energy and phase of condensed RRDGs are easily calculated using the von Neumann lattice as the ground state of the system. We assumed that the gas is a condensate in a pancake-shaped geometry and that the interaction energy per particle is smaller than the trap energy. The degree of freedom of the condensate in the *z*-direction was then frozen into the ground state of the harmonic oscillator in the *z*-direction. The centrifugal forces in the rapidly rotating system lead to a reduction in the density of the RRDGs, whereupon the system approaches the weak-interaction limit and falls into the LLL regime^8^.

We take the wave function Ψ_*q*_(***r***) in Eq. (4) as the ansatz of the ground state. The ansatz is a superposition of the coherent states in the LLL, *H*_0_Ψ_*q*_(***r***) = 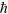
ΩΨ_*q*_(***r***). Within the Thomas-Fermi approximation, the ground-state energy of the system is determined using interaction energy 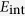:


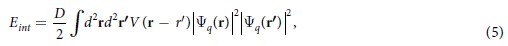
where *V*(**r**) is given in Eq. (2). Comparing *E*_int_ in the case of lattices with different geometric structures (labelled by the number *q* of flux quanta per unit cell), we can then find the phase diagram of vortex lattices in RRDGs for interactions with various blockade radii.

**Figure 5:**
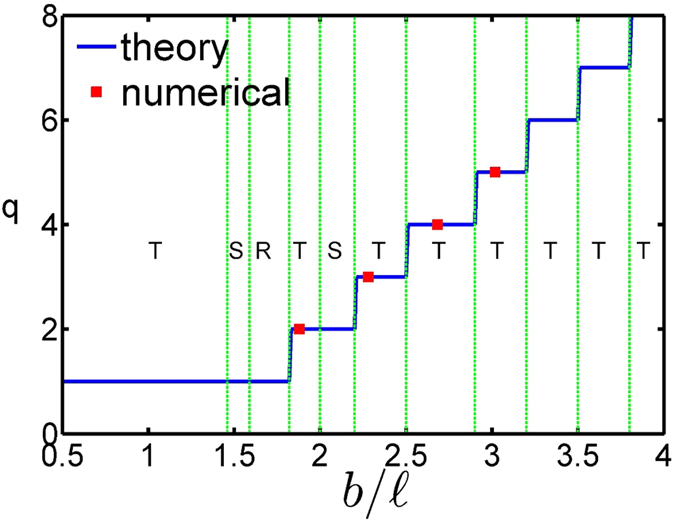
Phase diagram of triangular vN_*q*_ lattices. The horizontal and perpendicular axes represent the blockade radius and the number of flux quanta through a unit cell of vN_*q*_ lattices, respectively. **T**, **S**, and **R** denote triangular, square, and stripe lattices, respectively. The solid blue lines and red squares are the results obtained from ground-state energy calculations and numerical simulations of rapidly rotating gases with dipole interatomic interactions, respectively.

In the case with a fixed rotational frequency and blockade radius^14^,^15^,^16^, it was found that the vN_1_ lattices are stable when 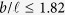. These lattices are listed as follows: triangular Abrikosov lattice 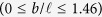, square Abrikosov lattice 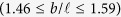, and stripe phase 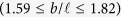. A phase diagram of energetically stable vortex lattices of RRDGs is presented in Fig. 5. The number of flux quanta *q* per unit cell in these lattices increases with *b*. In the regime of 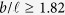, we identified the following sequence of vN_*q*_ lattices with *q* > 1: triangular vN_2_ lattice 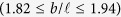 and square vN_2_ lattice 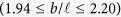. Due to the fact that 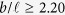, the triangular vN_*q*_ lattices represent the stable states in the regimes, as follows: vN_3_ lattices 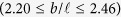, vN_4_ lattices 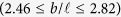, vN_5_ lattices 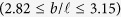, vN_6_ lattices 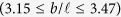, and vN_7_ lattices 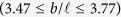.

vN_*q*_ lattices with *q* > 1 are energetically favorable in the regime of a larger blockade radius, the density and vortex distributions of which are presented in Fig. 2. Atoms in the vN_*q*_ lattices with *q* > 1 are clustered periodically and surrounded by vortices, which increase in number according to *b*. The potential of Eq. (2) has an equal-potential plateau with a width *b*. Bosonic atoms around this plateau would feel a uniform strength of atomic interactions and can accumulate together without too much energy cost. The number of clustered atoms around the plateau depends on the blockade radius *b*. The larger and smaller blockade radius produce the higher and lower numbers of clustered atoms, respectively. The quantum pressure associated with atoms becomes stronger as the number of clustered atoms increases in number according to *b*. As we know that the higher quantum pressure would squeeze a higher number of flux quanta into a unit cell, then the number *q* of flux quanta per unit cell increases in number according to *b*.

## Conclusions

In this study, we extended the theory of von Neumann lattices to rapidly rotating Bose gases. We applied von Neumann lattices to identify energetically stable vortex lattices in rapidly rotating gases with dipole interatomic interactions. Atoms in these lattices are clustered periodically and surrounded by vortices, which increase in number with an increase in the blockaded radius. The same result was also obtained in numerical simulations. We therefore conclude that von Neumann lattices, as generalized vortex lattices, can be physically realized using vortex lattices in rapidly rotating Bose gases with long-range interactions. We expect that the proposed theory will provide a starting point for the development of vortex-lattice models of greater sophistication. For example, we expect that von Neumann lattices are also related to the effects seen in the Landau-level mixing on vortex lattice structures^22^, the formation of vortices inside superfluid droplets^17^, and the structural phase transitions of vortex matter in two-component Bose-Einstein condensates^34^.

## Additional Information

**How to cite this article**: Cheng, S.-C. and Jheng, S.-D. Physical Realization of von Neumann Lattices in Rotating Bose Gases with Dipole Interatomic Interactions. *Sci. Rep.* **6**, 31801; doi: 10.1038/srep31801 (2016).

## Supplementary Material

Supplementary Information
