# Supplementary material for: Physical Realization of von Neumann Lattices in Rotating Bose Gases with Dipole Interatomic Interactions
Source: Sci Rep. 2016 Aug 22;6:31801. doi: 10.1038/srep31801 (PMC4992895; doi:10.1038/srep31801)
Supplement: Supplementary Information [file srep31801-s1.pdf]

Supplementary Information for "Physical Realization of von Neumann Lattices in Rotating Bose Gases with Dipole Interatomic Interactions"

Szu-Cheng Cheng[1] & Shih-Da Jheng[2]

[1]*Dept. of Optoelectric Physics, Chinese Culture University, Taipei 11114, Taiwan, ROC*

[2]*Institute of Physics, National Chiao Tung University, Hsinchu 30010 ,Taiwan, ROC*

In this study, the structure of vortex lattices of rapidly rotating Bose gases with dipole interatomic interactions (RRDGs) was numerically obtained by solving the Gross-Pitaeviskii equation. The initial state of the Gross-Pitaeviskii equation was obtained from one of the vN_q lattices. The state at a later point in time was obtained by applying the split-operator method to the time evolution of the wave function. The initial state of the Gross-Pitaeviskii equation was obtained from one of the vN_q lattices. We then let the state evolve in imaginary time by substituting $-it$ with t. Thus, the system gradually approached a steady state over time.

After finding the numerical results of vortex lattices in RRDGs, we then compare the density distributions of vN_q lattices with numerical density distributions. vN_q lattices are used to describe vortex lattices labelled by the number q of flux quantums per unit cell, where $q > 1$. vN_q is a generalized vortex lattice, which becomes an Abrikosov lattice when $q = 1$. The density distributions, $\rho_1(\mathbf{r})$, of vN_1 lattices are presented in Fig. S1. These distributions present the same characteristics as Abrikosov lattices.[1] vN_1 lattices possess only a single vortex in a unit cell; therefore, the quantum pressure from the centrifugal forces of vortices around a vN_1–lattice site is not sufficiently strong to cause the clustering of atoms. As a result, atoms are distributed uniformly except in

areas near the site of vortices. Thus, a vN_q lattice acts as an Abrikosov lattice when $q = 1$. This fact is not surprising considering that the physical phenomena are independent of the choice of gauge. vN_1 lattices are a representation of Abrikosov lattices in the symmetric gauge.

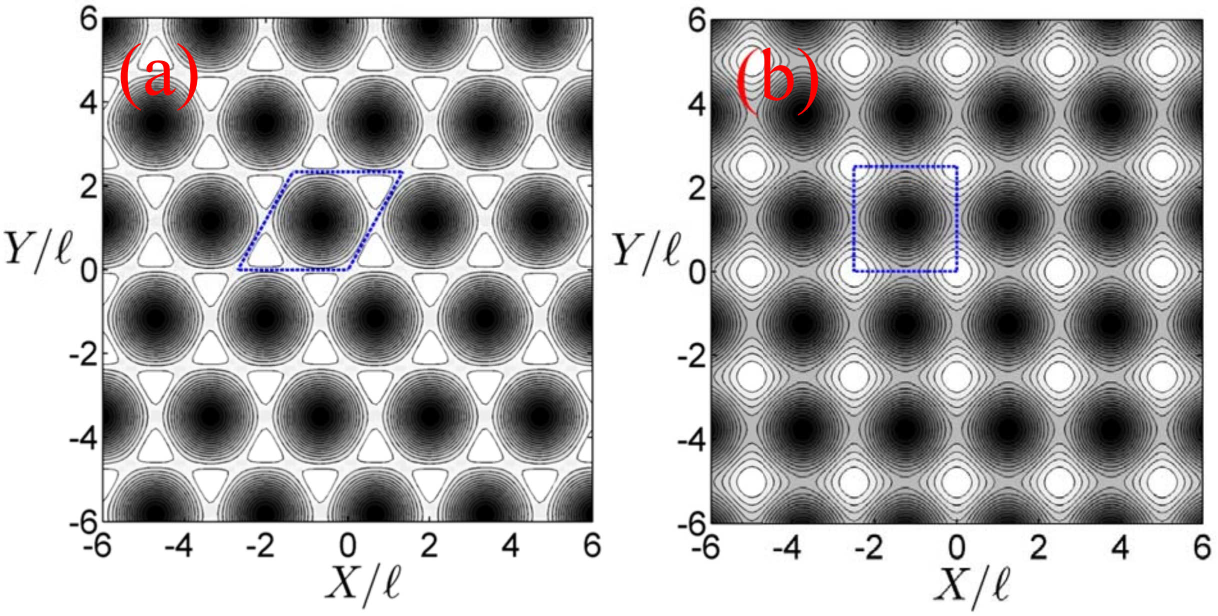

Figure S1: Contour plot showing particle density distribution in vN_1 lattices. The vN_1 lattices in (a) and (b) are triangular and square structures, respectively. The black area is the low-density regime indicating the location of a vortex and the bright area is the high-density regime. The blue dashed lines outline the area of a unit cell. Diagrams (a) and (b) present a flux quantum per unit cell in which the lattice constants are $d/\ell =$ 2.69 and 2.51, respectively.

In systems with contact interactions between particles, the ground-state energy of a vortex lattice is proportional to the inverse-participation-ratio β. In Table S1, we list several inverse-participation-ratio parameters β^T (for triangular structures) and β^S (for

square structures) based on vN_q lattices. It was found that parameter β^T of a triangular vN_q lattice is smaller than parameter β^S of a square vN_q lattice, when the number q of flux quantums per unit cell is fixed. Therefore, a triangular vN_q lattice has less energy and greater stability than does a square vN_q lattice. The inverse-participation-ratio parameter β also increases with the number q of flux quantums per unit cell. The triangular vN_q lattice with $q = 1$ presented the smallest β value where $\beta^T = 1.1596$. Thus, the triangular Abrikosov lattice or vN_1 lattice was shown to be more stable than other vortex lattices in physical systems with contact interactions between particles.

TABLE S1. Inverse-participation-ratio parameters β^T (for triangular structures) and β^S (for square structures) of von Neumann lattices. Symbol q indicates the number of flux quantums per unit cell.

q	β^T	β^S
1	1.1596	1.1803
2	1.3390	1.4248
3	1.6015	1.7015
4	2.0355	2.0598
5	2.5046	2.5155

The square vN_q lattice is stable in the regime $2.0 \le b/\ell \le 2.2$; however, Fig. S2 illustrates the density distributions of a square vN_q lattices in which $q =$ 2 to 5. The geometric structure of a vN_q lattice strongly affects the vortex distribution in a unit cell. Triangular vN_2 and vN_3 lattices respectively possess 2 and 3 single vortices per unit cell,

whereas the square vN_2 (vN_3) lattice has a vortex with double (triple) flux quantums per unit cell. Square vN_4 and vN_5 lattices respectively possess 4 and 5 single vortices per unit cell.

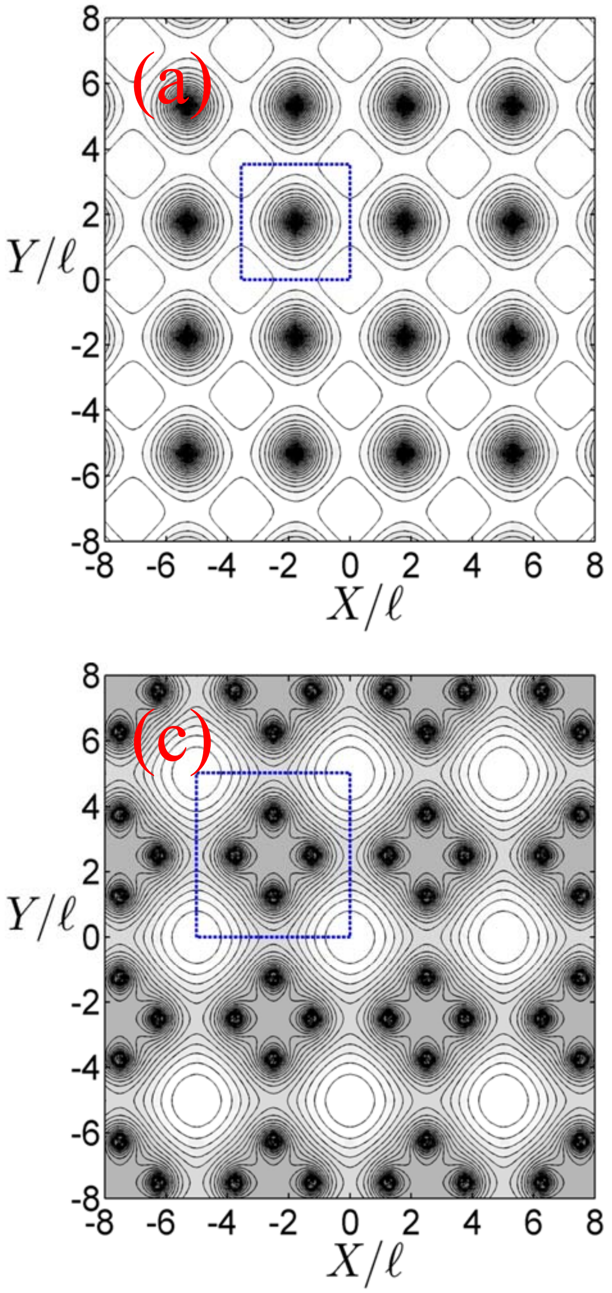

Figure S2: Contour plots of log-scaled particle density distributions of square vN$_q$ lattices. The low-density regime marked as a black area indicates the location of a vortex and the bright area is the high-density regime. The blue dashed lines outline the area of a unit cell. Diagrams (a) and (b) present the log-density distributions of square vN$_2$ and vN$_3$ lattices, respectively. Diagrams (c) and (d) present the log-density distributions of square vN$_4$ and vN$_5$ lattices, respectively. The lattice constants of square vN$_2$, vN$_3$, vN$_4$ and vN$_5$ lattices are $d/\ell = 3.54$, 4.34, 5.01 and 5.60, respectively.
